# Why do anti-inflammatory signals of bone marrow-derived stromal cells improve neurodegenerative conditions where anti-inflammatory drugs fail?

**DOI:** 10.1007/s00702-020-02173-3

**Published:** 2020-04-06

**Authors:** J. P. J. M. de Munter, J. Mey, T. Strekalova, B. W. Kramer, E. Ch. Wolters

**Affiliations:** 1 Neuroplast BV, Urmond, The Netherlands; 2 Division 3, Translational Neuroscience, School for Mental Health and Neuroscience, Maastricht University, Maastricht, The Netherlands; 3 Hospital Nacional de Parapléjicos, Toledo, Spain; 4 Department of Paediatrics, University Medical Centre (MUCM), Maastricht, The Netherlands; 5 Department of Neurology, UniversitatsSpital Zurich, Zurich, Switzerland

**Keywords:** Spinal cord injury (SCI), Amyotrophic lateral sclerosis (ALS), Human stromal/stem cells, Neuro-cells, Microglial activation, Methylprednisolone, Celecoxib, IL-1β, IL-6, GSK-3ß, IBA-1

## Abstract

Neurodegenerative disorders share the final degenerative pathway, the inflammation-induced apoptosis and/or necrosis, irrespective of their etiology, be it of acute and chronic traumatic, vascular and idiopathic origin. Although disease-modifying strategies are an unmet need in these disorders, lately, (pre)clinical studies suggested favorable effects after an intervention with bone marrow-derived stromal cells (bm-SC). Recent interventions with intrathecal transplantation of these cells in preclinical rodent models improved the functional outcome and reduced the inflammation, but not anti-inflammatory drugs. The benefit of bm-SCs was demonstrated in rats with an acute (traumatic spinal cord injury, tSCI) and in mice with a chronic [amyotrophic lateral sclerosis (ALS)-like FUS 1-358 or SOD1-G93-A mutation] neurodegenerative process. Bm-SCs, were found to modify underlying disease processes, to reduce final clinical SCI-related outcome, and to slow down ALS-like clinical progression. After double-blind interventions with bm-SC transplantations, Vehicle (placebo), and (non)steroidal anti-inflammatory drugs (Methylprednisolone, Riluzole, Celecoxib), clinical, histological and histochemical findings, serum/spinal cytokines, markers for spinal microglial activation inclusive, evidenced the cell-to-cell action of bm-SCs in both otherwise healthy and immune-deficient tSCI-rats, as well as wild-type and FUS/SOD1-transgenic ALS-like mice. The multi-pathway hypothesis of the cell-to-cell action of bmSCs, presumably using extracellular vesicles (EVs) as carriers of messages in the form of RNAs, DNA, proteins, and lipids rather than influencing a single inflammatory pathway, could be justified by the reported differences of cytokines and other chemokines in the serum and spinal tissue. The mode of action of bm-SCs is hypothesized to be associated with its dedicated adjustment of the pro-apoptotic glycogen synthase kinase-3β level towards an anti-apoptotic level whereas their multi-pathway hypothesis seems to be confirmed by the decreased levels of the pro-inflammatory interleukin (IL)-1β and tumor necrosis factor (TNF) as well as the level of the marker of activated microglia, ionized calcium binding adapter (Iba)-1 level.

## Introduction

Neurodegenerative disorders are becoming increasingly prevalent and are a growing burden on the population worldwide. Acute neurodegenerative disorders are caused by trauma or vascular problems, leading to apoptosis and inflammation. Though many molecular and genetic causes are thought to serve as predisposing or disease-propagating factors, the underlying pathogenesis in chronic neurodegenerative disorders such as amyotrophic lateral sclerosis (ALS) is in most cases still obscure. Recent discoveries in these diseases, though, have demonstrated the presence of inflammation propagating substrates, and trials with several potential immune-modulating therapies provided increasing evidence that primary induced apoptosis followed by secondary inflammation are heavily involved in the pathogenesis not only in acute but also in chronic neurodegenerative diseases. Although steroidal and non-steroidal anti-inflammatory drugs (N)SAIDs, with their anti-inflammatory effects, as well as various neurotrophic factors, with their pro-survival signaling mechanisms, in the past had been proven to be effective in attenuating neuronal death in many in vitro and in vivo models of neurodegeneration, all larger phase II/III trials with both (N)SAIDs and/or various neurotrophic factors, so far, did bring equivocal and/or worse outcomes (Gilgun-Sherki et al. 2006; Hernan et al. 2006; McGeer and McGeer 2007; Schwartz and Ziv 2008; Schwartz and Shechter 2010; Bracken 2012; Ling et al. 2016). Maybe wrong timing of administration, nonselective inhibition of COX-2 or Rho-A, sub-optimal dose in target site, or limited penetration to the brain through the blood–brain barrier here may have played a role. Differences between rodent models and humans aside, perhaps the most confounding factor might be that the point(s) of action might be downstream of the pathophysiological process and retrospective in terms of neuronal death induction. Neuroprotection via pro-survival signaling also might not adequately annihilate the continuing pathological insult or might be too late to reverse the demise of compromised neurons (Tang 2017).

Unfortunately, monitoring inflammation by identifying microglia-produced cytokines as biomarkers to help in the diagnosis, to predict the progression, and to target key immune factors in the various neurodegenerative processes is still a challenge. The translation of cytokines as a biomarker in clinical practice is further hampered by intra-individual variation, environmental factors, genetic background, disease stage, and anatomical onset of motor neuron impairment (Hu et al. 2017; McCauley and Baloh 2019; Moreno-Martinez et al. 2019a, b). Even age and gender play a role, and the different pro- and anti-inflammatory cytokines along the disease progression, therefore, should be further studied to understand its time point activation and its relation to other molecular and clinical mediators in neurodegeneration to finally provide a better monitoring of disease progression (Moreno-Martinez et al. 2019a).

Various genetic and environmental factors underly neurodegeneration, the result from activated inflammasomes, responsible for the activation of inflammatory responses removing cell debris, wastes and pathogens via phagocytosis. Inflammasomes are key signalling platforms that detect pathogenic microorganisms and sterile stressors, and that activate highly pro-inflammatory cytokines. In terms of molecular pathogenesis, neurodegenerative disorders share a negative contribution of non-neuronal cells (immune cells, glial cells) expressing and activating inflammasomes as a significant commonality (Lewis et al. 2012; Orsini et al. 2015).

Microglia, the resident immune cells in the CNS, comprise an entire spectrum of phenotypes that span the range from deleterious to regulatory to remodeling effects. Although microglial activation provides a defense against injury and infection, chronic or excessive activation is considered to be detrimental and has been implicated in many neurodegenerative and psychiatric disorders (Hammond et al. 2019). In response to their environment, microglia are able to rapidly change morphology and function (London et al. 2013). Gene expression analyses led to the identification of homeostatic (state 1 microglia) as well as “disease-associated” microglial phenotypes (DAMs) (Keren-Shaul et al. 2017). As DAMs are not associated with the disease etiology, but rather with a general response program that is involved in clearance of the protease-resistant misfolded and aggregated proteins, one still rather prefers the name “reactive” microglia. Out of the enormous variety of reactive microglia, two main types of reactive microglia have been defined: the classically activated (cytotoxic) (state 2) microglia that affect neuronal survival and that secrete pro-inflammatory cytokines, tumor necrosis factor (TNF), interleukins-1β and 12 (IL-1β, IL-12), interferon-gamma (IFN-γ), and nitric oxide inclusive, the so-called interferon response microglia (IRMs); and the alternatively activated (cytoprotective) microglia that express genes involved in innate immune response, thus supporting an anti-inflammatory response and preventing classical microglial activation, called activated response (state 3) microglia (ARMs). ARM-response overlaps with the DAM-response described by Keren-Shaul (Keren-Shaul et al. 2017), but these cells are not necessarily disease associated and might be considered part of the normal evolution of microglia in healthy aging. At odds with the original DAM-response description is also the heterogeneity in the ARMs. Alternatively, activated microglia secrete interleukin-4 (IL-4), interleukin-13, or transforming growth factor β (Hammond et al. 2019). Distinct ‘reactive’ microglia signatures can be used to better understand microglia function and to identify and manipulate specific subpopulations in health and disease. The ionized calcium-binding adapter marker (Iba)-1, which is upregulated in reactive microglia (IRM and ARM), is often used to identify these cells in general (Sala-Frigerio et al. 2019).

In acute neurodegenerative disorders, the classical activated microglia response (IRM) is caused by primary insult-induced necrosis, whereas in chronic disorders, ongoing activation is elicited by a genetic and/or environmental-driven abnormal accumulation of misfolded proteins, mitochondrial dysfunction, oxidative stress and/or inflammation, all processes which reinforce each other (Lim et al. 2007; Ganguly et al. 2017). In ALS, as with other neurodegenerative diseases, the degeneration is a complex interplay between multiple pathogenic cellular mechanisms such as oxidative stress, mitochondrial dysfunction, impaired axonal transport, excitotoxicity, protein aggregation, endoplasmic reticulum stress, neuroinflammation, abnormal RNA processing, non-neuronal cells, and target muscle contribution (Mancuso and Navarro 2015).

Activated cells proliferate and form dense clusters around the cell bodies of injured neurons (Ramirez et al. 2011). These classically reactive phenotypes of intrinsic microglia and/or monocyte-derived macrophages produce a pro-inflammation signal cascade by the secretion of various cytotoxic factors, pro-inflammatory signaling molecules, and the expression of immune molecules, TNF and other inflammatory cytokines inclusive. They also increase their expression Iba-1. Once classically activated, state 2 microglia start to express glycogen synthase kinase-3β (GSK-3β), what they normally not do (Tang et al. 2015), thus further promoting both innate and adaptive immune responses (Wang et al. 2011; Beurel et al. 2015). GSK-3β, though, has an interesting paradoxical effect: a high dose initiates a pro-apoptotic effect during mitochondrial-mediated intrinsic apoptosis but in a low dose, it induces an anti-apoptotic effect during death receptor-mediated extrinsic apoptosis. Low doses of GSK-3 inhibitors, therefore, provide a feasible means to counteract excessive inflammation and induce neuroprotective actions in chronic neurodegenerative conditions, among them amyotrophic lateral sclerosis (Mazzardo-Martins et al. 2012). Activation of microglia is a hallmark of brain pathology, and reactive microglia are especially thought to be involved in neuroinflammatory responses (Ito et al. 1998). As a consequence, the environment around the damaged neurons becomes toxic, thus further enhancing the degeneration (Subramaniam and Federoff 2017).

Once the inciting event has been adequately resolved, a lower production of pro-inflammatory cytokines will polarize these cells, switching them from state 2 microglia (IRMs) into state 3 microglia (ARMs) that express genes involved in innate immune response, facilitating phagocytosis of cell debris and misfolded proteins, promoting tissue repair, and supporting neuronal survival by neurotrophic factors (Khalid et al. 2017).

However, when the pathogenic stimulus cannot be adequately cleared, chronic inflammation develops with a persistent IRM response that can cause unintended injury to local tissues. Thus, chronic neurodegeneration is facilitated by the lack of neurotrophic growth factors and by the continued production of cytotoxic by-products of a pro-inflammatory response (Hooten et al. 2015). The type, location and connections of the necrotic cell populations are leading in the (variable) clinical expression of these neurodegenerative processes.

## Anti-inflammatory drug interventions in neurodegeneration

As discussed above, neurodegenerative diseases share strong neuroinflammatory multi-pathway components, which are at least in part responsible for the continuing cell death. Pharmacological treatments, therefore, should intend to reduce this inflammatory response. Curiously, so far, interventions with anti-inflammatory drugs in patients suffering from these disorders did not result in successful treatments (Gilgun-Sherki et al. 2006; Hernan et al. 2006; McGeer and McGeer 2007; Schwartz and Ziv 2008; Schwartz and Shechter 2010; Ling et al. 2016). In patients suffering acute neurodegeneration such as spinal cord injuries, in which secondary inflammatory processes are mainly responsible for final clinical outcome, initially it was suggested that methylprednisolone (MP) did significantly reduce disability, though this could not be confirmed in later studies, and side effects appeared to outweigh any beneficial effects (Hurlbert 2000; Bracken 2012). Also, in patients suffering from chronic neurodegenerative disorders, Alzheimer disease, Parkinson disease and amyotrophic lateral sclerosis (ALS) inclusive, anti-inflammatory drugs were found to be ineffective (Fondell et al. 2012; Collins and Bowser 2017; Khalid et al. 2017; Crisafulli et al. 2018). Activation of microglia and astrocytes with increased levels of pro-inflammatory serum and CSF cytokines IL-1β, IL-6, IL-8, TNF and vascular endothelial growth factor (VEGF) is considered the hallmark in ALS (Ciervo et al. 2017; Hu et al. 2017; Morello et al. 2017; Crisafulli et al. 2018). Interventions with corticosteroids and/or other anti-inflammatory drugs such as the COX-2 inhibitor celecoxib, which causes the reduction of the brain levels of inflammatory cytokines TNF and IL-1β (Osman et al. 2016), however, were found ineffective in experimental ALS-like animals and/or ALS patients (Galbiati et al. 2012; Collins and Bowser 2017; Crisafulli et al. 2018). Apart from an eventual, subtle, inconsistent increase of survival time in ALS which might be seen in some patients after an intervention with riluzole (Miller et al. 2012) and/or edaravone (Yoshino and Kimura 2006), disease-modifying interventions, for instance, adequate inflammasome-targeted strategies, are still an unmet need in most neurodegenerative disorders (Voet et al. 2019). Further, growth factors may play a potential role in facilitating functional recovery in degenerative neurons (Shruthi et al. 2017).

## Interventions with BM-derived stromal cells in neurodegeneration

Although interventions with anti-inflammatory drugs in experimental neurodegenerative animal studies have not resulted in successful clinical trials, a more recent approach to treat such disorders, in both experimental animals and humans, with implanting bone marrow-derived stromal cells (bm-SCs), seems very promising. Such transplants supposedly modulate the immune system in both acute (Deda et al. 2008; Martinez et al. 2017; Tsai et al. 2018; Cofano et al. 2019; Jin et al. 2019) and chronic (Mazzini et al. 2016; Ciervo et al. 2017; Gashmardi et al. 2017; Sykova et al. 2017; Cizkova et al. 2018; Garbuzova-Davis et al. 2018; Oh et al. 2018; Gugliandolo et al. 2019) neurodegenerative disorders in experimental animals as well as in patients. Our own experiments with human bm-SC in experimental animal models of both an acute (traumatic spinal cord injury; tSCI) (de Munter et al. 2019a; Romero-Ramirez et al. 2020) and a chronic neurodegenerative disorder (amyotrophic lateral sclerosis; ALS) (de Munter et al. 2019b) were fully in line with these findings. In these studies, fresh bm-SCs specimen were manufactured into standardized preparations for intrathecal application, to bring naïve stem cells into the environment where neuroinflammation and degeneration are ongoing. Hereto, these specimens were reduced in volume after positive depletion of erythrocytes, monocytes and lymphocytes and negative selection of untouched stem cells (Neuro-cells: patent WO2015/059300A1).

We explored the effect of an intrathecal transplantation with bm-SCs (4 × 10^5^ CD34^+^ cells) in both T-cell-deficient and immune-competent Wistar rats, 1 day after a traumatic spinal cord injury, resulting in a complete paraplegia (de Munter et al. 2019a; Romero-Ramirez et al. 2020). Bm-SCs were found to be free of side effects, to significantly protect for SCI-related mortality, and to improve natural motor recovery (Basso et al. 1995) compared to placebo-treated animals, in the following weeks (de Munter et al. 2019a). When compared to an acute 48-h intra-peritoneal high-dose methylprednisolone (MP) application (Romero-Ramirez et al. 2020), bm-SC again significantly improved locomotor functions (Basso et al. 1995) and restored body weight. Bm-SC-treated rats also never evidenced SCI-associated neuropathic pains during testing of mechanical nociception (von Frey), as sporadically seen in SCI rats treated with placebo and/or MP. In those experiments, there was no rejection of the transplants, and there were no adverse events.

The effects of an intrathecal intervention with bm-SCs (5 × 10^5^ CD34^+^ cells) were also studied in two different experimental ALS-like mice models. As the etiology of ALS is still unclear, animal models are based on gene mutations which are found in familial cases: Cu–Zn superoxide dismutase gene SOD1, C9ORF72, PGRN, TBK1, TARDBP, and FUS genes (Freischmidt et al. 2015; Al-Chalabi et al. 2016; Lutz 2018). Still asymptomatic 10-week-old FUS (1-358) (Shelkovnikova et al. 2013) and 12-week-old SOD1 (G93A) mutant mice (de Munter et al. 2019b) were treated with bm-SC transplantations before developing a progressive loss of motor functions, muscle atrophy and weight loss due to a fast degeneration of spinal motor neurons with denervation in the following weeks, thereby representing a relevant preclinical model for amyotrophic lateral sclerosis (ALS). Conforming with the previous studies (Uccelli et al. 2012; Boido et al. 2014; Ciervo et al. 2017), intrathecal bm-SC transplantations in FUS (1-358) and SOD1 (G93A) mutant ALS-like mice were found to significantly induce disease-modifying effects; they significantly delayed cachexia, weight loss and motor dysfunction, as well as muscle atrophy and the loss of spinal lumbar motor neuron as seen in placebo-treated transgenic mice. Interventions with riluzole and/or celecoxib at the same (pre-symptomatic) age in these animals, however, failed to slow down the development of these ALS-like symptoms in the mutant mice.

## Mode of action of BM-derived stromal cells

The mode of action of bone marrow-derived stromal cells was originally postulated to be related to cellular integration by leveraging the plasticity of the stromal/stem and progenitor cells for the replacement of lost neural tissue. In addition, the mechanism was also considered to relate indirectly via cellular interactions which stimulate secretion of neurotrophic factors as well as factors affecting the immune response by modulating T- and B-cell activities thereby decreasing apoptosis of neural cells and inflammatory responses (Ruppert et al. 2018). Mesenchymal stem cells (MSCs) and hematopoietic stem cells (HSCs) are the main stem cells sourced by bone marrow (Kucia et al. 2005). Both stem cell types are capable of differentiating into spindle neuron-like cells (Koshizuka et al. 2004; Sigurjonsson et al. 2005; Ye et al. 2011). Further, after harvesting, HSCs differentiate easily into pro-inflammatory cells increasing inflammation. These cells, though, can be kept in their naïve status at the same time when MSCs were present (Le Blanc and Ringden 2005).

As stem cells only sparsely pass intact blood–brain barriers (BBB) and/or CSF–brain barriers, it is most probably that eventual effects of bm-SCs are not reached by cell replacement but might rather be effectuated by communicators, signaling proteins that freely pass those barriers, though. If given intrathecally, the bm-SCs are trapped in the cerebrospinal fluid and disappear within a couple of weeks completely (Engelhardt and Sorokin 2009; Redzic 2011; Abramowski et al. 2016). The bm-SCs are decision-making cells that coordinate their operations with their immediate environment (Fischbach et al. 2013; Caplan 2017). They act cell-to-cell using all kinds of communicators, such as extracellular vesicles (EVs) and soluble factors such as cytokines (including chemokines, interferons, interleukins, lymphokines, and tumour necrosis factor), growth factors, and mitochondria transfer. EVs are small, membrane-bound nanoparticles that can be released from most, if not all cells, and that can carry functionally active cargo (proteins, nucleic acids) that has been shown to modify the recipient cells physiology to react in a paracrine and endocrine manner (Yanez-Mo et al. 2015; Zhang et al. 2016; Harting et al. 2018; Ruppert et al. 2018). The EVs–cargo which comprises DNA, RNA, protein, and lipids reflects the physiological as well as the pathophysiological state of a cell (Abels and Breakefield 2016). EVs are emerging as a promising tool for therapeutic delivery owing to its favorable intrinsic features of biocompatibility, stability, stealth capacity, and the ability to overcome natural barriers (Dostert et al. 2017; Shahjin et al. 2019). In other studies, bm-SC-EVs were found to exert immune-suppressive effects by enforcing ARM macrophage polarization and stimulating T-cell induction as well as producing neurotrophic factors and anti-inflammatory cytokines, as a dedicated reaction of environmental vesicles or cytokines from degenerating, malfunctioning cells (Dostert et al. 2017; Cizkova et al. 2018; Harting et al. 2018; Kim et al. 2018; Wang et al. 2018; Beers and Appel 2019; Shahjin et al. 2019). This hypothesis, though, is still a hypothesis, and recently, the possibility that bm-SCs one way or the other might donate healthy mitochondria to neurons that harbor dysfunctional mitochondria has also been raised to explain their positive effects (Babenko et al. 2015).

To further address the question why immune-modulating bm-SCs strategies are more successful than anti-inflammatory drugs in human neurodegenerative disorders, we want to summarize the different cytokines and proteins with the relevant pathway and want to discuss a compilation of all findings from three preclinical studies, looking after the effects of an intervention with bm-SC in in SCI and ALS-like animal models (de Munter et al. 2019a, b; Romero-Ramirez et al. 2020). The following cytokines and proteins were studied in the mentioned preclinical experiments:

### TNF

Tumour necrosis factor (TNF) is important to maintain immunity and cellular homeostasis creating a balance between cell survival, apoptosis, and necroptosis. TNF regulates the generation of reactive oxygen species (ROS) and reactive nitrogen species (RNS), and this ROS/RNS signaling plays an important role in activating and controlling inflammatory conditions (Blaser et al. 2016). Further TNF initiates the MAPK signaling pathway and the NF-kappaB signaling pathway, both involved in apoptosis and necroptosis (Wrzodek et al. 2013).

### IL-1β

Interleukin-1β is a pro-inflammatory cytokine and a mediator of neuronal injury. Experiments with interleukin-1 cause an impaired cerebral blood flow (Murray et al. 2015). Mechanistically, it seems that interleukin-1β is also correlated with the caspase pathways, thus initiating apoptosis (Spinello et al. 2019).

### IL-6

Interleukin-6 is a cytokine that is released by proteolytic cleavage and is involved in the regulation of the immune system and inflammation within the central nervous system. IL-6 binds to an IL-6 receptor, and then associates with a dimer of the ubiquitously expressed gp130 receptor subunit, which initiates intracellular signaling. Interleukin-6 can bind to the membrane of cells (liver and leucocytes) and initiate a classical signaling, which is regenerative and anti-inflammatory, though the soluble form can bind with a co-protein to nearly all cell types and initiate trans-signaling, which is inflammatory and pro-apoptotic (Rose-John 2012). IL-6 signaling activates downstream signaling pathways such as Janus kinases/signal transducers and activators of transcription (Jak/STAT), the phosphatidylinositol 3-kinase cascade and the mitogen-activated protein kinase cascade through gp130 homodimer formation (Garbers et al. 2012).

### Iba-1

Iba-1 is a marker of reactive microglia (Imai and Kohsaka 2002). Microglial activation presumably involves the FGF2/FGFR1 (fibroblast growth factor and receptor) pathway as can be seen when this pathway is blocked (Zou et al. 2019). Microglia play an important role in modulating inflammation, and especially the polarized (ARM) microglia contribute to a more regenerative environment. This was confirmed, as a matter of fact, by the microglial expression of arg1, CD206 and CCR2 (Roszer 2015) in our experiment (Fig. 2). However, he activation of microglia might also be reached by the modulating effects of Il-6 and TNF (Chu 2013; Schaper and Rose-John 2015).

### GSK-3β

Glycogen synthase kinase-3 is responsible for maintaining selective intracellular phosphorylation of many substrates. This enzyme is involved in all kinds of roles in multiple signaling pathways. The upregulation of GSK-3β expression in T cells is seen as pathogenic in autoimmune diseases (Beurel et al. 2013). Blocking GSK-3 may bring down disease progression in neurodegenerative diseases by decreasing inflammation and apoptosis, and providing a cell-protective environment (Morales-Garcia et al. 2013).

**Fig. 1:**
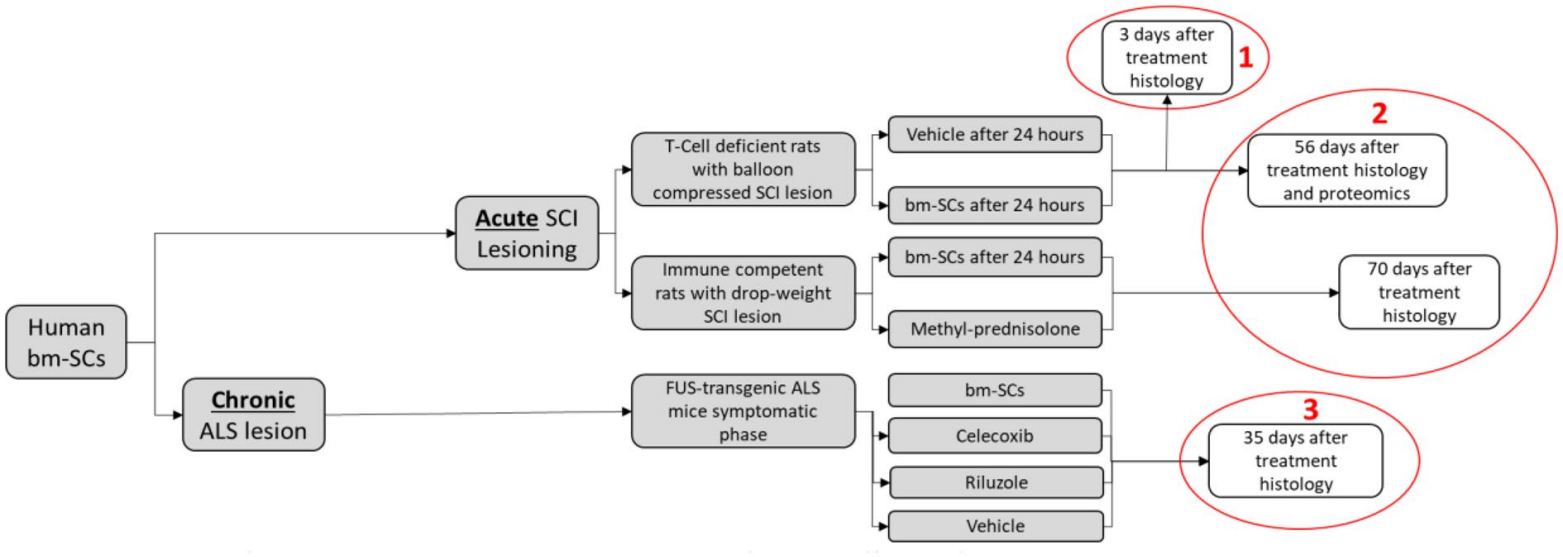
Overview of the three preclinical studies, looking after the effects of an intervention with the same bm-SCs in acute and chronic degenerative diseases, providing the different time points of tissue sampling for histology

Figure 1 presents an overview of the time axis of these three studies in which we compared the histological effects of bm-SC with the effects reached after matched interventions with placebo (vehicle), methyl prednisolone, riluzole and/or Celecoxib in SCI-lesioned and ALS-like experimental animals, at different time points after intrathecal application (1, 2, and 3). The findings will be put into the perspective of the hypothesis that stem cells are decision-making cells and that they adapt to their environment.

**Fig. 2:**
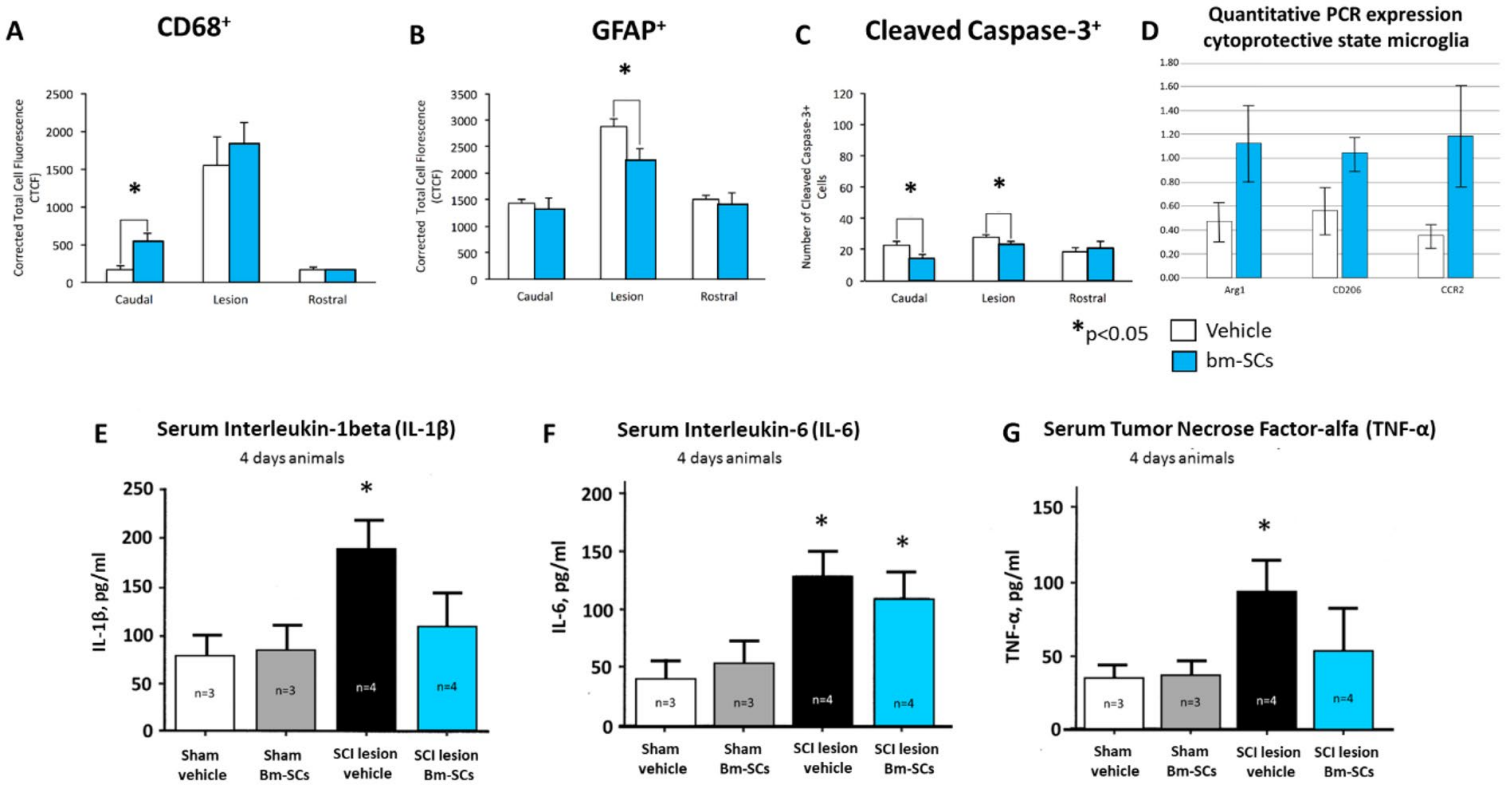
Expression of microglia (**a**), astroglia (**b**), and apoptosis (**c**) caudal, central and rostral of the SCI lesion, and the quantified polarization from IRM to ARM microglia (**d**) 3 days after the interventions with bm-SCs or vehicle in T-cell-deficient balloon compression-induced spinal cord injured rats. **e** The serum concentration of interleukin-1β, **f** the serum interleukin-6, and **g** the serum TNF levels in the sham and SCI-lesioned rats treated with vehicle or bm-SC (de Munter et al. 2019a)

At the time point marked with number 1 in the red circle (Fig. 1), 3 days after the administration of the intrathecal intervention with bm-SCs or vehicle and 4 days after the initial lesioning, histology was performed of the spinal tissue in both balloon compression-induced SCI-lesioned and sham-lesioned immune-deficient rats (caudal, central, and rostral of the eventual lesion). Findings are displayed in Fig. 2. Naïve bm-SCs applied 24 h after the lesioning straight to the cerebrospinal fluid of a SCI injured rat were able to polarize IRMs into ARMs, to decrease astrocytes in the lesion, to protect neurons for apoptosis in the lesion and to decrease serum pro-inflammatory interleukins (IL-1β, IL-6, and TNF) when compared to the vehicle-treated animals. The focus of the bm-SC cells was to “detox” the cerebrospinal fluid and save precious neurons in an acute neurodegenerative condition (de Munter et al. 2019a).

**Fig. 3:**
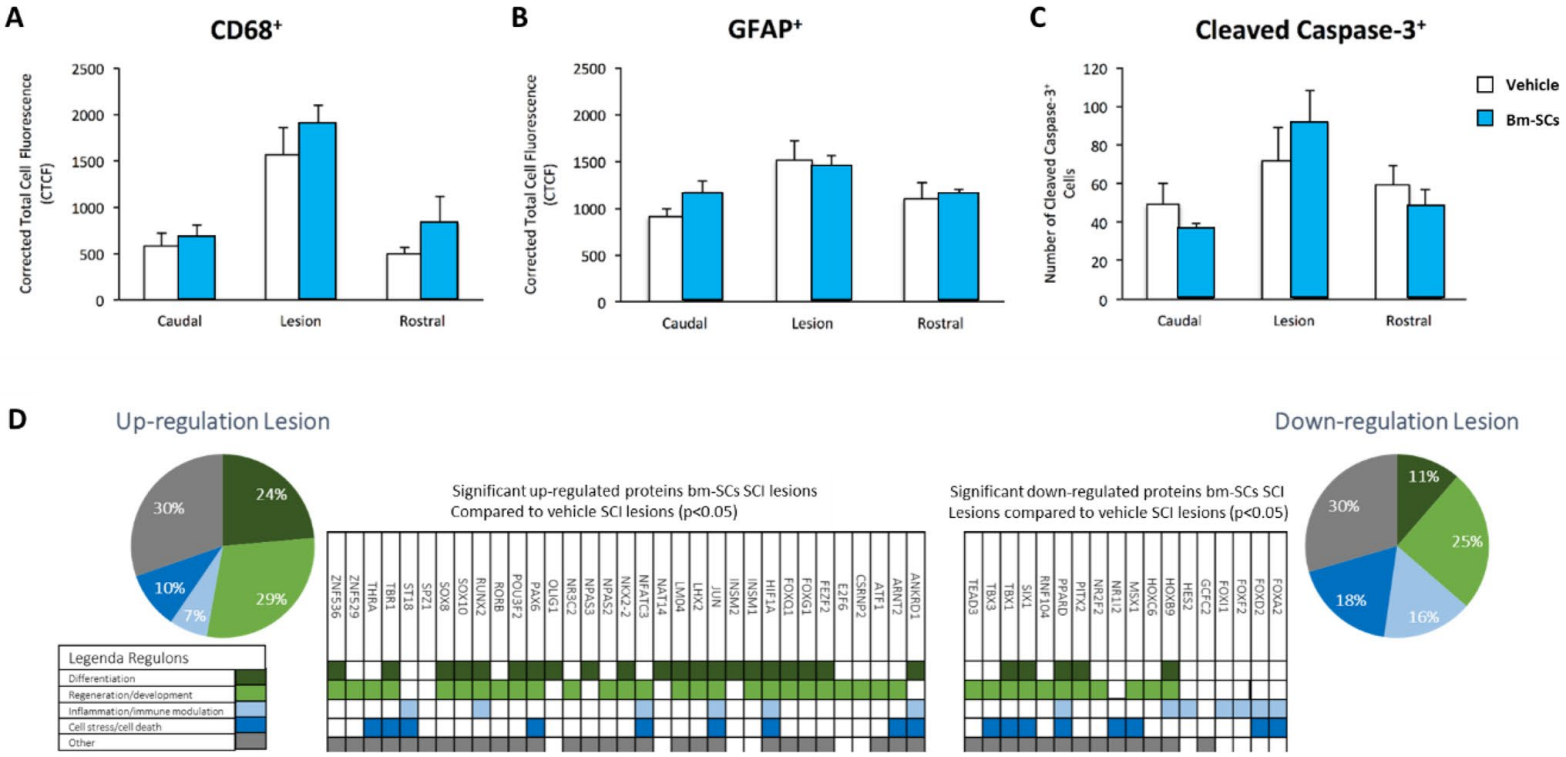
Expression of microglia (**a**), astroglia (**b**) and apoptosis (**c**) caudal, central, and rostral of the SCI lesion in the spinal cord of animals killed 56 days after the intervention with bm-SCs or vehicle, in T-cell-deficient balloon compression-induced spinal cord-injured rats. In **d**, the significant (*p* < 0.05) up- and down-regulated proteins of the analyzed lesioned spinal tissue are presented. Protein expression in the injured tissues of the vehicle-treated animals are displayed in the baseline (de Munter et al. 2019a)

At the time point marked with number 2 in the red circle (Fig. 1), 56 days after the administration of the intrathecal intervention with bm-SCs or vehicle and 57 days after the initial balloon compression-induced lesioning in immune-deficient rats, histology was performed of the spinal tissue (caudal, central, and rostral of the lesion) and lesion tissue was prepared for proteomics to investigate the up- and downregulation of different regulons. Findings at this time point are displayed in Fig. 3.

**Fig. 4:**
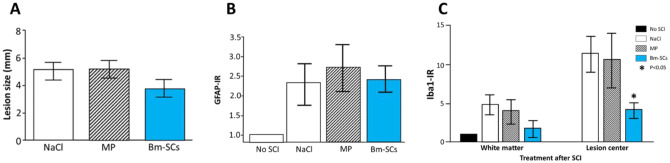
Lesion size as evaluated with cleaved Caspase-3 (**a**), expression of astroglia (**b**) and Western blot Iba-1 (**c**) in the spinal cord tissue [data were normalized to Iba1-IR in the white matter of the not-lesioned (No SCI) rats] in immune competent rats after a drop-weight-induced SCI lesion, 70 days after an intervention with methylprednisolone (MP), vehicle (NaCl) or human stem cells (bm-SCs) (Romero-Ramirez et al. 2020)

At the time point also marked with number 2 in the red circle (Fig. 1), 70 days after the administration of the intervention with bm-SCs, methylprednisolone or vehicle, and 71 days after the initial drop-weight lesioning of otherwise healthy rats, lesion site, expression of astroglia and Western blot Iba-1 in the spinal lesion site were investigated. Findings are displayed in Fig. 4.

**Fig. 5:**
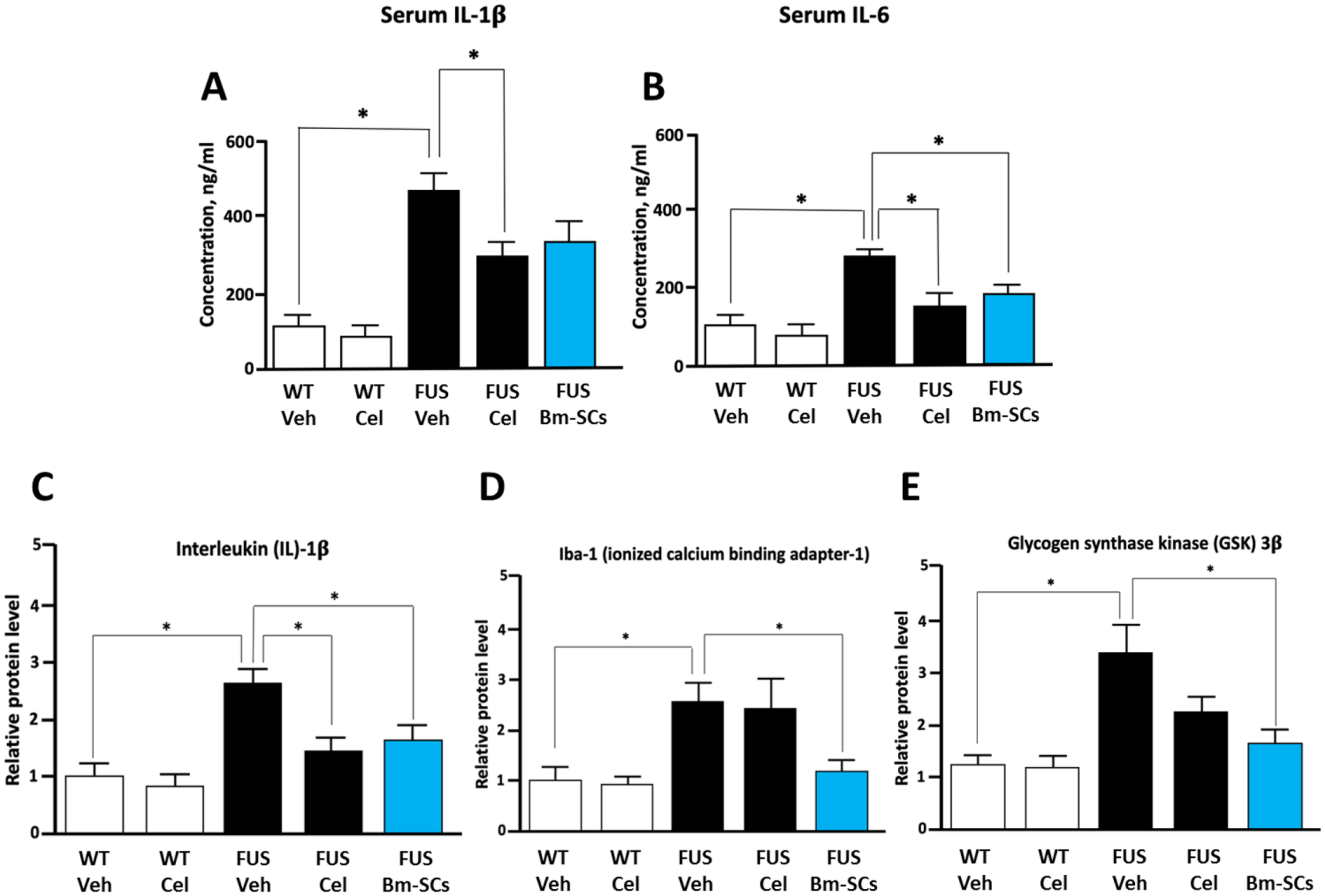
Overview of the ELISA serum IL-1β (**a**) and IL-6 (**b**) levels, and the Western blot staining of spinal IL-1β (**c**), Iba-1 (**d**) and GSK-3β (**e**) levels in wild-type (WT) and FUS-tg mice treated with vehicle, riluzole, celecoxib and bm-SCs as established 35 days after these interventions in the at-that-time asymptomatic animals (de Munter et al. 2019b)

Finally, at the time point marked with number 3 in the red circle (Fig. 1), 35 days after the application of vehicle, bm-SCs, riluzole or celecoxib in still asymptomatic ALS-like FUS-mutated mice, ELISA serum and spinal Western blot histology were performed (de Munter et al. 2019b). Findings are displayed in Fig. 5.

As shown in Fig. 2, 3 days after the intervention with the vehicle, the devastating effects of the SCI lesioning in the vehicle-treated rats were mainly found due to a persistent neuroinflammatory response, as reflected by the increased expression of microglia in IRM mode, as well as the increased astrogliosis and apoptosis, when compared to the animals treated with bm-SC (de Munter et al. 2019a).

The results reached at the various time points after the interventions with bm-SCs are summarized in Table 1.

**Table 1:** Summary of the effects of interventions with intrathecally applied bm-SC in experimental animal models for both acute (SCI) and chronic (ALS-like) neurodegenerative disorders

Intrathecal application 24 h after SCI lesioning	Cytokine TNF ↓ (*p* < 0.05) in serum and spinal tissue
	Cytokines IL-1β ↓ (*p* < 0.05) and ↓ IL-6 (trending)
	Iba-1 ↓ (*p* < 0.05) in serum and spinal tissue
	Polarize cytotoxic macrophages 2 (IRMs) into cytoprotective macrophages 3 (ARMs) apoptotic/pro-inflammatory proteins ↓ (*p* < 0.05)
	Regenerative/differentiating proteins ↑ (*p* < 0.05)
Intrathecal application in still aymptomatic ALS-like FUS-tg mice (see Fig. 5)	Cytokine IL-1ß ↓ and IL-6 levels ↓ in serum and spinal tissue (*p* < 0.05)
	Iba-1 ↓ (*p* < 0.05) in serum and spinal tissue
	GSK-3β ↓ (*p* < 0.05) in spinal tissue

Fifty-six days after the interventions, histological studies of the lesioned tissues in the bm-SC and vehicle-treated SCI-lesioned animals did not establish any significant difference in the expression of microglia, astrocytes, and apoptosis anymore (see Fig. 3). A comprehensive proteomic profile of the lesioned spinal tissue showed various changes in the up- and downregulation of the protein expression when compared with the findings at baseline (vehicle treated animals), which were set to 100%. The most important findings were the downregulation of pro-inflammatory proteins and the upregulation of the proteins involved in axonal and cellular regeneration.

As might be appreciated in Fig. 4, 70 days after the intervention with vehicle, methylprednisolone or bm-SC in drop-weight-induced SCI-injured, otherwise healthy, rats, the absence of any significant difference in histological findings as established 56 days after the intervention with bm-SC and vehicle-treated SCI-lesioned immune-deficient rats, as displayed in Fig. 3, could be confirmed in immunocompetent rats. The lesion size in the animals treated with bm-SCs was somewhat smaller than in the rats treated with vehicle and methylprednisolone, but not significantly. An interesting finding, though, was the significantly lower expression of Iba-1 in the lesion site of the animals treated with bm-SCs, but not in the animals treated with methylprednisolone and/or vehicle (Romero-Ramirez et al. 2020) confirming the hypothesis that bm-SCs reach anti-inflammatory effects by different means than pharmacological interventions.

In these animals, apoptosis, as evaluated with activated caspase-3, was found significantly reduced in the ventral horns as well as axonal pathology in the ascending dorsal columns when compared to vehicle and/or MP-treated rats (Romero-Ramirez et al. 2020) (Fig. 4).

The most straightforward answer to the initial question about the mode of action of bm-SCs seems to be the multi-pathway approach of bm-SCs in contrast to the single pathway approach of (N)SAIDS. An alternative explanation could be the activation of a still not clear pathway more upstream of the inflammatory cascades. Bm-SCs might be seen as decision-making cells that coordinate their operations with their immediate environment (Fischbach et al. 2013; Caplan 2017). Their cell-to-cell communication here may play a major role and future research must elucidate the content of stem cell-secreted EVs in modulating inflammatory environments.

**Fig. 6:**
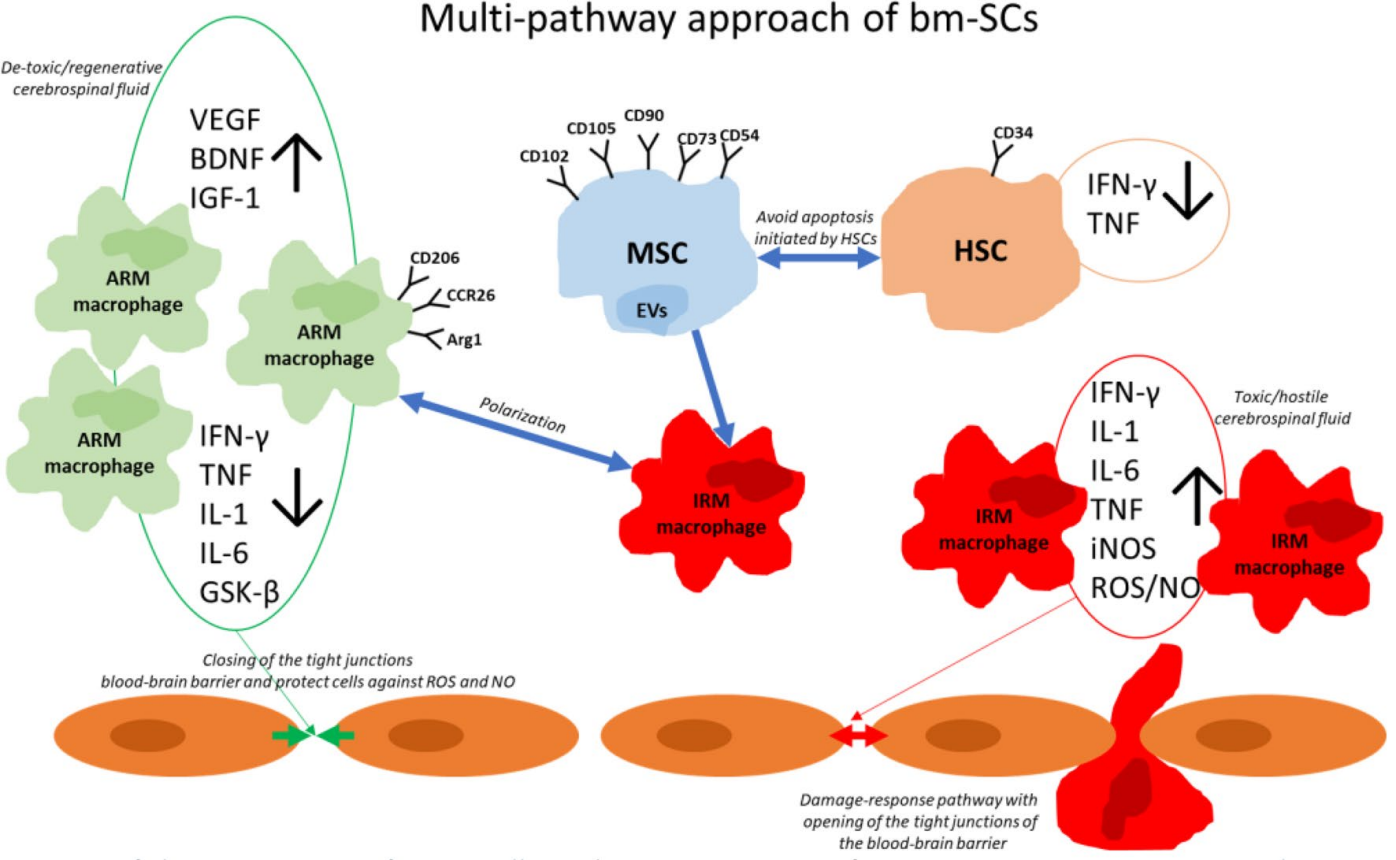
An overview of the interaction of stem cells in the maintenance of a regenerative non-toxic cerebrospinal fluid. The MSCs by cell-to-cell communication polarize the IRM macrophages into ARM macrophages. The ARM macrophages decrease the pro-inflammatory cytokines and enhance the growth factors. ARM macrophage gene expression for CD206, CCR26, and Arg1. *ARM* activated response microglia, *BDNF* brain-derived neurotrophic factor, *EVs* extracellular vesicles, *GSK-β* glycogen synthase kinase-3 beta, *HSC* hematopoietic stem cell, *IGF-1* insulin-like growth factor-1, *IFN-γ* Interferon-gamma, *IL-1* interleukin-1, *IL-6* interleukin-6, *iNOS* inducible nitric oxide synthase, *IRM* interferon response microglia, *MSC* mesenchymal stem cell, *NO* nitric oxide, *ROS* reactive oxygen species, *TNF* tumor necrose factor, *VEGF* vascular endothelial growth factor)

In summary, bm-SCs’ immune-modulating properties, including their paracrine cytokines with their polarizing effect on IRMs, attack more and different mechanisms than the individual anti-inflammatory drugs, as also suggested by our proteomic findings in the SCI rats (de Munter et al. 2019a). Despite the evidence that inflammation is critical in both SCI and ALS, treatment with various anti-inflammatory drugs (including Rho-A, COX-2 and TNF inhibitors, steroids, cyclophosphamide, cyclosporine, cytochrome-C inhibitors, and caspase-reducing drugs) seems to fail to clinically significant modify SCI and/or ALS symptomatology and/or pathology in humans (Hurlbert 2000; Bracken 2012; Collins and Bowser 2017; Crisafulli et al. 2018), unless the result of insufficient dosage or bioavailability, delayed time points of delivery, and/or or the taking-over of the eventually inhibited pathway by other single or downstream pathways. By the way, the bm-SC-induced immunomodulatory and regenerative effects might also be mediated when the stem cells quickly pass on their effects to resident cells (Eggenhofer et al. 2014). Figure 6 illustrates the multi-pathway approach of bm-SCs.

As for the application route of stem cells in patients suffering from neurodegenerative disorders, in our opinion, bm-SC might be best implanted intrathecally. Intravenous application ends up with most bm-SCs stuck in lung and liver (Fischer et al. 2009), and the number of engrafted bm-SCs in the central nervous system will be minimal due to the lack of ability to easily cross the blood–brain barrier by these cells (Cerri et al. 2015). On top of this, repeated intrathecal bm-SC transplantations proved to be safe and feasible, and are found most promising for the treatment of patients with neurological diseases (Pan et al. 2019).

## Conclusion

Evidence is provided for bm-SCs to

1. significantly improve survival and accelerate natural motor recovery in rats suffering balloon compression and/or weight-induced spinal cord injuries with a complete paraplegia in combination with reduced apoptosis, whereas methylprednisolone failed to do so;
2. significantly delay the onset of ALS-like symptoms in pre-symptomatic FUS- and SOD1-transgenic mice, whereas riluzole and celecoxib failed to do so.

These effects may be initially reached by bm-SCs preventing the neurodegeneration-induced inflammatory reaction with sharply increased serum/spinal Il-1β and TNF levels, later on, having passed their effects to resident cells, in combination with bringing down the pro-inflammatory and pro-apoptotic degeneration-induced effects, as evidenced by their reduction of raised GSK-3β and the conversion of IRM microglia into ARM microglia. As (N)SAIDs are clinically ineffective in these acute and chronic neurodegenerative conditions, bm-SCs here might reach their effects by reducing specifically the increased GSK-3β and Iba-1 protein levels in the affected central nervous system. It is possible that bm-SCs might reach these effects by acting on other than myeloid cells. Finally, the fact that bm-SCs can adapt their activity depending the environment where they are in, justifies the manufacturing/processing approach to keep these cells in a naïve condition before entering the disease arena.
